# Heterogeneity in patient diagnostic pathways: an example from contrast-enhanced ultrasound diagnostic scans for focal liver lesions

**DOI:** 10.1186/1756-0500-7-199

**Published:** 2014-03-31

**Authors:** Adam B Smith, Alexandra Filby, Louise M Carr

**Affiliations:** 1 York Health Economics Consortium Ltd. University of York, Market Square, Heslington York YO10 5NHUK

**Keywords:** Contrast-enhanced ultrasound, MR, CT, Imaging modalities

## Abstract

**Background:**

The UK’s National Institute for Health and Care Excellence (NICE) has recommended contrast-enhanced ultrasound (CEUS) for the characterisation of focal liver lesions where the results of standard unenhanced ultrasound are inconclusive. A further recommendation is for CEUS to replace other imaging modalities. However, little is currently known about the diagnostic pathways in the National Health Service (NHS) followed by patients with potential liver lesions. The aim of this study was to identify the diagnostic pathways for a number of representative hospital trusts and record the clinicians’ views on patient experiences of these processes through a series of semi-structured interviews with UK clinicians (radiologists and sonographers) (N = 7). This study was undertaken in the broader context of a larger research project where the overarching research question is focused on patient preferences for CEUS and other imaging modalities, and how these impact on patient quality of life (QOL).

**Results:**

The results from the semi-structured interviews with UK clinicians revealed that there is a great deal of heterogeneity in diagnostic pathways followed by patients with potential liver lesions which differ both within and between hospitals. In terms of the patient experience, the clinicians believed that a combination of the more patient-friendly ultrasound process, and the fact that scan results are given to patients in 80-90% of cases on the day, as well as the problems inherent to other scan modalities (claustrophobia, anxiety) would lead to patients preferring ultrasound compared with other imaging modalities (CT or MR). However, current clinical practice means that patient choice is virtually non-existent.

**Conclusions:**

The significant variation in diagnostic pathways across the NHS will require further standardisation through local agreements if contrast-enhanced ultrasound is to replace other imaging modalities in characterising focal liver lesions in line with NICE Diagnostics Guidance. The gradual development of patient choice of modalities may necessitate a change of practice in radiology processes.

## Background

Contrast-enhanced ultrasound (CEUS) using SonoVue® contrast agent (sulphur hexafluoride microbubbles) has been shown to accurately differentiate between malignant and benign focal liver lesions [1], characterise focal liver lesions [2,3] and liver metastases [4]. Furthermore, serious adverse events are very rare with this technology and are usually mild [5,6].

In August 2012, the UK’s National Institute for Health and Care Excellence (NICE) issued guidance on the use of SonoVue® contrast agent for contrast-enhanced ultrasound imaging of the liver [7]. The NICE guidance recommended the use of SonoVue® for a number of diagnostic processes: 1) the characterisation of incidentally detected focal liver lesions in adults where an unenhanced ultrasound has been inconclusive, and 2) in patients undergoing surveillance for cirrhosis or metastatic disease for whom contrast-enhanced (CE) computed tomography (CT) or magnetic resonance imaging (MR) is not clinically appropriate, accessible or acceptable (to the patient). Furthermore, the latter is indicated where a contrast agent is required for further diagnosis and an unenhanced US scan is unsatisfactory or inconclusive.

**Figure 1:**
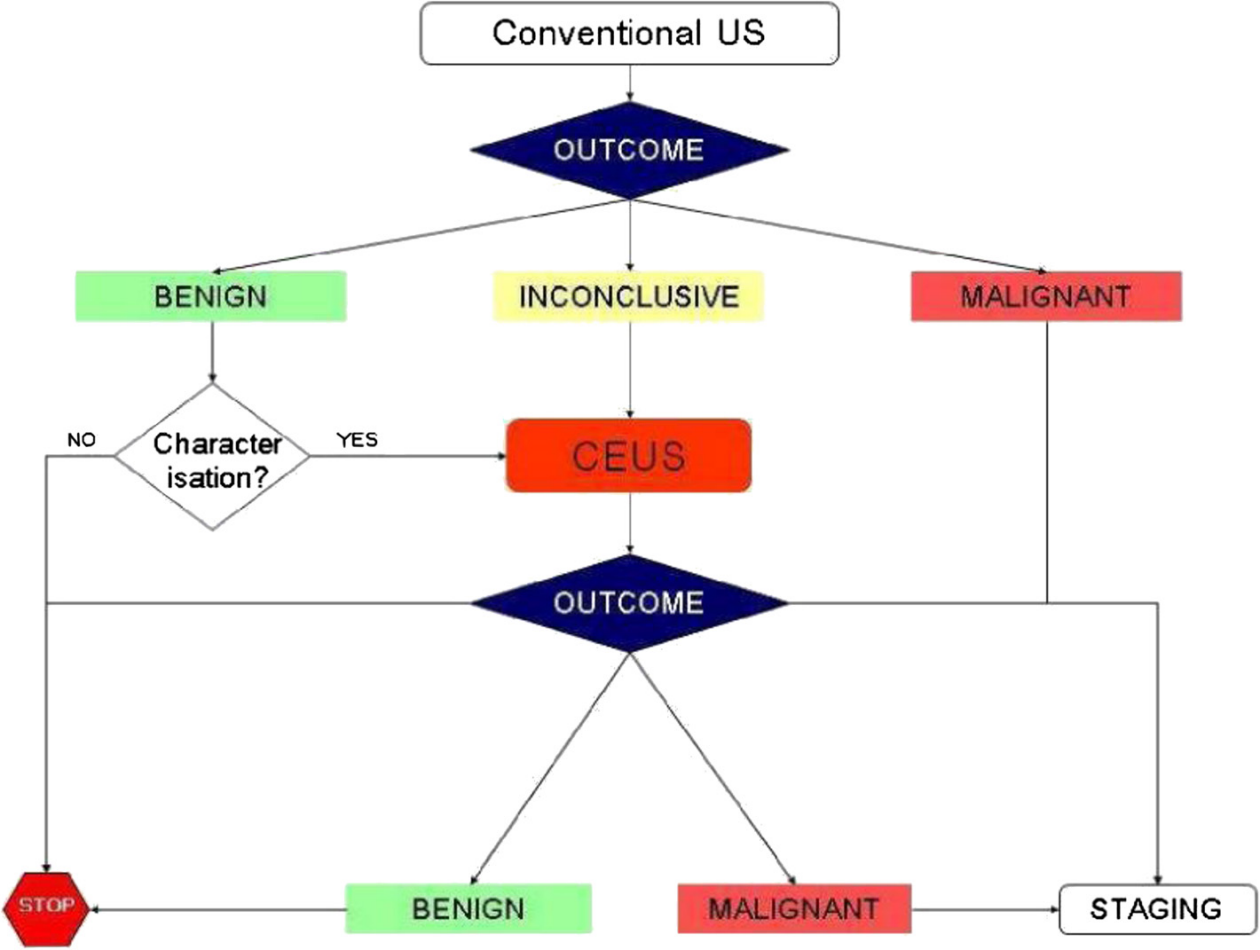
Diagnostic pathway for liver imaging with contrast-enhanced ultrasound as a replacement for contrast-enhanced CT/contrast-enhanced MR.

**Figure 2:**
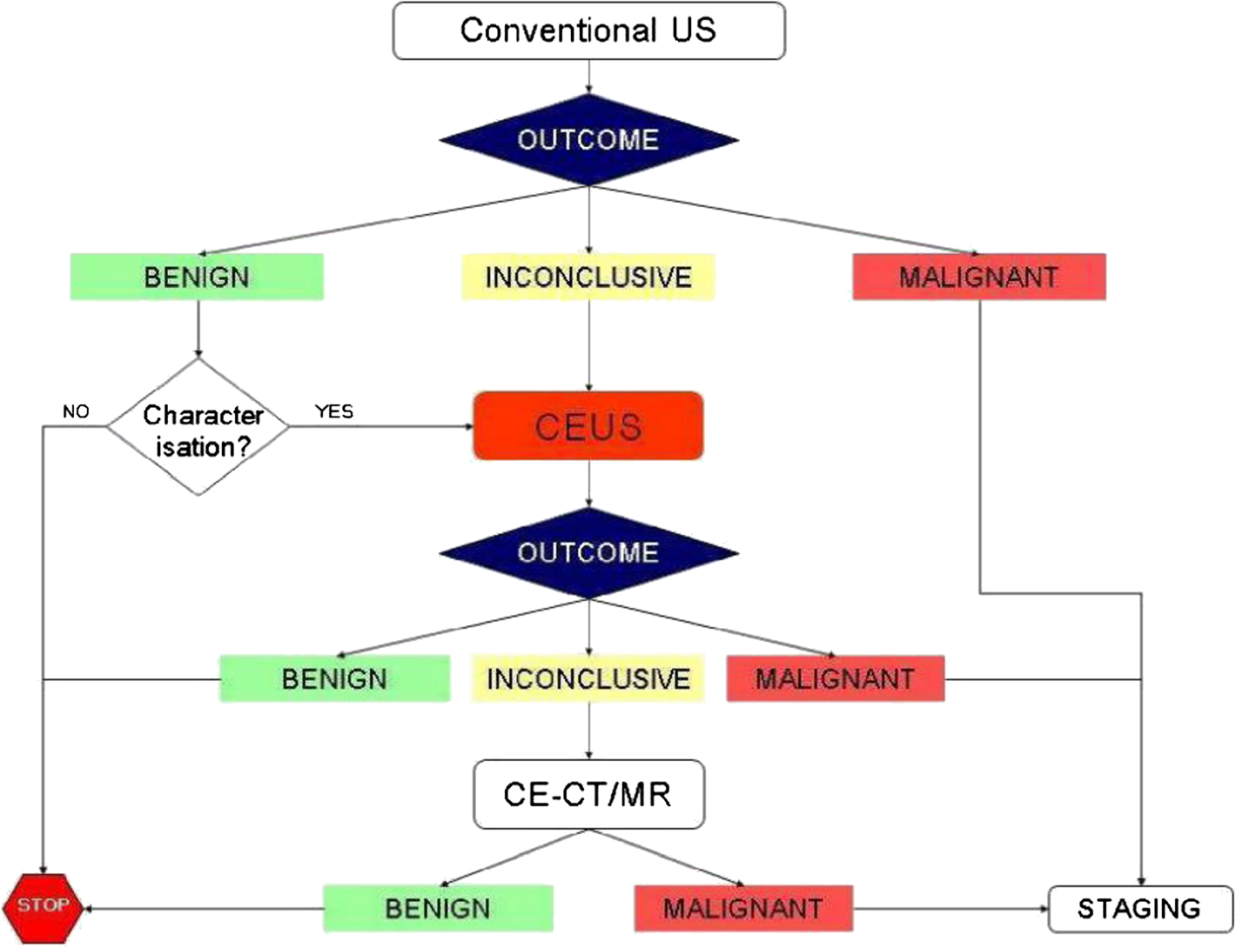
Diagnostic pathway for liver imaging with contrast-enhanced ultrasound as a triage test to reduce the use of contrast-enhanced CT/contrast-enhanced MR.

The NICE guidance suggests that CEUS may be used in the diagnostic pathway to replace contrast-enhanced CT (CECT) and contrast enhanced MR (CEMR). The NICE guidance also suggests that CEUS could be used as a “triage step” in order to minimise the use of these two technologies, however, the available data only allowed CEUS to be included as a replacement for CECT and CEMR. The potential diagnostic pathways for these two options are shown in Figures 1 and 2. However, a scoping search identified that little is currently known about the diagnostic pathways that patients follow when undergoing unenhanced/enhanced imaging for focal liver lesions.

This study was undertaken in the broader context of our role as an External Assessment Centre (EAC) for the NICE Medical Technologies Evaluation Programme (MTEP) along with the Regional Medical Physics Department at the Freeman Hospital (Newcastle Upon Tyne NHS Foundation Trust UK)^a^. The overarching research question is focused on patient preferences for CEUS and other imaging modalities, and how these impact on patient QOL, based on the recommendations for further research in the diagnostics guidance issued by NICE [7]:

Research is recommended on patient preferences, and their impact on quality of life, for contrast-enhanced ultrasound and other imaging modalities. Ideally such research should compare all appropriate imaging modalities in the same patient group [Section 7, 7.2].

The results of this research will help inform the development of a survey instrument to detail patient experiences of imaging scans, as well as a discrete choice experiment to capture patient preference. Therefore, the aim of this study was to produce an overview of clinical practice with regards to diagnostic scans for incidental focal lesions (CEUS/CECT/CEMR). In particular, we were interested in recording the diagnostic pathway followed by the patient from the point of referral (by the GP or as an inpatient or outpatient), through to the scan appointment (and process), and outcome, as well as detailing clinicians' views on their patients' experiences of this process, and how this could impact on patients’ quality of life (QOL).

## Methods

### Sample

The study used purposive sampling in recruiting the participants: Bracco SpA (Milan, Italy, manufacturer of SonoVue®) provided a list of hospitals and UK clinicians (radiologists and sonographers) that use SonoVue® and who had consented for their details to be made available to third parties. None of the clinicians were contacted before approval had been sought and provided by Bracco. Seven clinicians were approached based on the high volumes of SonoVue® used at their centres, of these, four responded and were interviewed. A further three contacts were provided by our partners at Newcastle Upon Tyne NHS Foundation Trust. In total, seven interviews were carried out. As this study was a service evaluation no research ethics or NHS R & D approval was required (http://www.hra.nhs.uk/).

### Design

Semi-structured interviews were held with clinicians either in person or over the telephone. Use was made of the NICE Guidance (DG5) to identify pathways, diagnostic and referral processes associated with the scanning modalities. Furthermore, a preliminary review of the literature had been undertaken to identify questions and concerns of patients undergoing scans (e.g. claustrophobia, adverse events). Both of these were employed to develop a set of questions which was used to guide the interviews with clinicians (Additional file 1). The interviews were scheduled to last an hour and were conducted by two interviewers (AF, ABS). Detailed notes of the interview were independently taken by both interviewers, and were reviewed after the interview. Any omissions and/or errors were corrected and a penultimate draft of the interview was then transcribed. This was sent to the clinician concerned for comments and feedback.

## Results

A total of 7 interviews were held with senior clinicians at consultant radiographer or radiologist grade, all of whom had significant experience in using SonoVue®. The clinicians were employed at different NHS trusts across England (North East, East Anglia, West Yorkshire and London). The interviews lasted on average around 30 minutes. The initial interviews tended to take slightly longer as the interviewers familiarised themselves with the questions (approximately 45 – 60 minutes).

**Figure 3:**
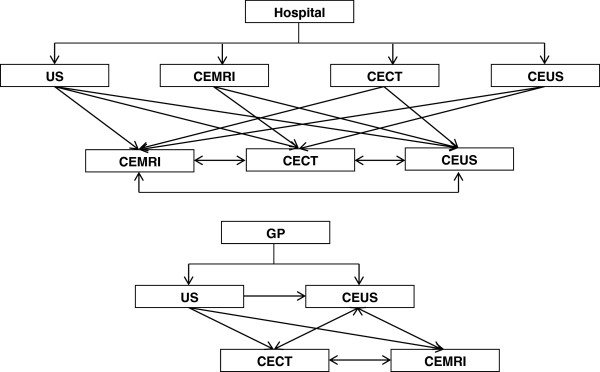
The possible diagnostic pathways followed by patients.

There were large reported variations in clinical practice both within and between different hospitals and NHS trusts, and there were differences (within hospitals) between individual clinicians. For instance, some hospitals / trusts had local clinical guidelines in place in terms of referral and scanning pathways, for others, these decisions were based on individual clinical judgement (see Figure 3). This scenario is further complicated by the fact that contrast-enhanced ultrasound scans may be provided by sole clinicians or a team of clinicians and not all clinicians are trained to use SonoVue.

The diagnostic pathway between and within hospitals varied at several different points. Patients were referred through the GP or as an inpatient/outpatient (including those on screening programmes). Waiting times for a CEUS or standard ultrasound (US) appointment varied considerably across trusts from between 1–2 weeks to 6 weeks (maximum waiting period recommended by the NHS). The median waiting time for this sample was 2 weeks, which corresponds to the UK median waiting time for diagnostic scans [8]. Lengthier waiting times are to be expected particularly for GP referrals, as the GP would in most instances not know that a focal liver lesion was present. One clinician reported that patients may also receive the CEUS on the same day as another scan. The differences in wait times depended on several factors, including: the availability of trained staff and hospital process (some hospitals were willing to ‘absorb’ the cost of performing a CEUS while only charging for the standard US that the patient had been referred for, in hospitals that were not willing or able to do this the patient was to be referred back to their practitioner). The wait time for results also varied between and within hospitals with many patients receiving their results immediately. The majority of clinicians (5/7, 71%) reported that patients would be informed of their results on the day of the scan if the lesion was benign. These clinicians noted that patients would not be informed immediately if the lesion was malignant. However, one clinician noted that patients would be told of a malignancy if in his/her opinion the patient would be able to cope with this information.

Contrast-enhanced ultrasound is currently used to characterise a known lesion or as a diagnostic process. The procedure times were approximately the same across the scanning modalities at around 20–30 minutes (as reported by all 7 clinicians). Patients were unlikely to experience more than two of the scanning modalities (i.e. they tended to experience standard US plus one of the contrast enhanced investigations) according to the clinicians interviewed.

Adverse reactions or side-effects to CEUS were virtually nonexistent: one clinician reported the incidence of adverse reactions as <0.1% (another clinician reported two cases in 10 years of practice; however, neither of these was attributable to the ultrasound contrast agent). In contrast to this, all clinicians (N = 7) noted that MR/CT may induce anxiety and feelings of claustrophobia and the contrast agent used in CT scans was associated with side effects, such as, a metallic taste, the feeling of bladder incontinence, nausea and/or a rash. An increased risk of an adverse reaction to the contrast agent used in CEMR was reported compared to that used in CEUS. Furthermore, the ultrasound scan process was described as a more patient-friendly and informal process than CT or MR scans. The clinician would be present in the room to talk to the patient during the process and a friend or family member could be present.

Ultrasound was described as having a shortened referral pathway waiting time with the majority of clinicians providing the results for benign cases immediately after the scan. However, practice varied on this point with some clinicians (N = 2) informing inpatients of the results 24–72 hours after the scan, or sending the results back to the referrer (hospital or GP). For MR/CT, patients did not receive their results immediately. These were sent to the referring clinician introducing further delays (and potentially more anxiety) for the patient (see Table 1). The shortened waiting time for the results (ultrasound) was reported to lead to less patient anxiety. Both of these results are features of CT and MR scans which produce multiple and more detailed images than ultrasound requiring more time to be reviewed.

**Table 1:** Summary length of time to receive results

**Modality**	**Lesion benign**	**Lesion malignant**
CE-US (SonoVue)	-Immediate	Varies considerably by hospital:
	-Within 24 hours if inpatient, or	-Less than 1 week
	-Within 1 week, if results sent to GP	-Between 2 – 8 weeks
MR/CT		Varies considerably (>than for CE-US)

Currently, patients do not have a choice of which scanning modality they receive. This was based virtually always on clinical judgment (although patients do have the choice of refusing a scan). However, in an isolated case a single patient was able to choose the scanning modality (ultrasound) following a refusal to undergo another MR scan.

## Discussion

The clinicians reported significant heterogeneity between and within hospital trusts in terms of the scan process with the patient diagnostic pathway varying significantly. These initial results from the semi-structured interviews suggest that ultrasound has little impact on patient quality of life QOL. In contrast to this, the MR/CT process (scanner) and time to results may impact on QOL (e.g. anxiety), however, this may be transient and will be influenced by the outcome of the scan. The different processes across hospitals, as well as the inherent problems with MR/CT (e.g. anxiety and claustrophobia) are likely to impact on QOL, however the ability to detect this is going to be negatively affected as any impact on QOL may be overshadowed by the “noise” created through the variation in clinical practice (diagnostic accuracy and the actual outcome of the scan as a confounding variable). The clinicians suggested that patients (if given a choice) would prefer ultrasound as there are fewer symptoms/side-effects, and less anxiety induced by the clinical process (results in most cases are provided at the time of the scan) compared to the MR/CT scanners.

The NICE Guidance [7] document for SonoVue® suggests that CEUS could be employed either as a replacement for CECT or CEMR. The NICE guidance also suggests (but does not include in its recommendations) that CEUS could be used as a “triage step”. It appeared from the interviews that, in practice, CEUS was being used as a triage step to reduce the amount of CT or MR scans (i.e. it was not only being used when a CT or MR was not clinically appropriate, accessible or acceptable to the patient). The findings of this study suggest that, given the heterogeneity in diagnostic pathways apparent across hospitals, this may be difficult to implement across the UK’s NHS without further standardisation of diagnostic pathways.

The study was potentially limited by the small sample size, however, the clinicians interviewed were from Trusts of various sizes, geographic locations and with varying practices and therefore should be representative of the range of practices currently in place in the NHS. Furthermore, given the seniority of those interviewed we were able to capture a broader view of diagnostic imaging processes. However, it should be noted as a further potential limitation that the study only focused on the patient pathways for practitioners with considerable experience of using the technology, which may have introduced bias.

In terms of providing a basis for further research, the results from the clinician interviews suggest patients’ experiences of the imaging modalities will primarily be impacted by the inherent differences between ultrasound and MR/CT scans, including the scan experience and the variations in diagnostic pathway. The clinicians interviewed reported that CEUS has virtually no side-effects, has shorter waiting times for appointments and the results can be provided to patients immediately in the majority of cases, thereby reducing any anxiety. Furthermore, the ultrasound process itself, in contrast to the other modalities, should not induce any anxiety or claustrophobia. However, the investigation itself may be very stressful to patients who want to be reassured their lesion is benign. It may therefore be posited that any differences in patient experience and QOL across the modalities would be explained by these facts alone (and influenced by the outcome of the scan, which is common across the modalities). In other words, all things being equal, given a similar diagnostic outcome the patient experience will be more positive for ultrasound compared to the other modalities. These results will help inform the development of a survey instrument to detail patient experiences of ultrasound scans, as well as a discrete choice experiment to capture patient preference.

## Conclusions

There is a degree of heterogeneity in respect of diagnostic pathways for the characterisation of focal liver lesions meaning that any attempts to introduce contrast-enhanced ultrasound as a replacement or triage step for other imaging modalities may require further standardisation.

## Endnote

^a^This part of the EAC is undertaking a prospective audit detailing the incidence of incidental focal liver lesions.

## Abbreviations

CE: Contrast-enhanced; CECT: Contrast-enhanced computerised tomography; CEMR: Contrast-enhanced magnetic resonance imaging; CEUS: Contrast-enhanced ultrasound; CT: Computerised tomography; DG: Diagnostics guidance; MTEP: Medical Technologies Evaluation Programme; QOL: Quality of life; US: Ultrasound.

## Competing interests

The authors declare that they have no competing interests.

## Authors’ contributions

ABS, AF and LC designed the survey; ABS and AF conducted the interviews. All authors contributed to the manuscript.

## Supplementary Material

Additional file 1 Questions for Clinicians. Click here for file
