# Supplementary material for: Heterogeneity in patient diagnostic pathways: an example from contrast-enhanced ultrasound diagnostic scans for focal liver lesions
Source: BMC Res Notes. 2014 Mar 31;7:199. doi: 10.1186/1756-0500-7-199 (PMC4230804; doi:10.1186/1756-0500-7-199)
Supplement: Additional file 1 — Questions for Clinicians. [file 1756-0500-7-199-S1.doc]

**Additional File 1 - Questions for Clinicians**

Background: NICE has recommended SonoVue to characterise incidentally detected focal liver lesions in adults in whom an unenhanced ultrasound is inconclusive. **To check with clinician:** Our understanding is that these lesions may be picked up during abdominal scanning for other purposes than specifically looking for lesions.

 What is your area of speciality? Is it in one imaging modality?

 Do you have any experience of using Sonovue?

 For patients that are referred on to have an MRI or CT scan to look at the liver, will

it be **CE**MRI and **CE**CT?

 How many patients are referred (roughly) for CRMRI, CECT and CE-ultrasound?

 If so, are there any differences between contrast media used in the

CEUS/CEMRI/CECT? [administration]

 What contrast media is used? Brand names?

 Depending on clinicians area of speciality – Are patients normally asymptomatic

when referred on for further imaging? Can you give an estimate of how many have symptoms and what these are likely to be?

 Are you aware of any side effects of adverse events associated with each imaging

type?

 Based on your experience with patients, what do you think they perceive to be

negative/positive about each type of scanning modality?

 Do you know what the waiting time or from referral until an appointment for each

scanning type?

 Do you know how long the patient is likely to wait, from arrival at the hospital, until

scan takes place?

 What is the approximate duration of each scanning modality when looking at the

liver?

 If a patient has an inconclusive ultrasound, would a CEUS be performed in the

same appointment or would they have to return to the hospital/clinic?

 Would you be willing to review our (draft) patient survey?

 Are there other colleagues we could approach?
